# Convergent evolution in human and domesticate adaptation to high-altitude environments

**DOI:** 10.1098/rstb.2018.0235

**Published:** 2019-06-03

**Authors:** Kelsey E. Witt, Emilia Huerta-Sánchez

**Affiliations:** 1 Cell and Molecular Biology, University of California—Merced, 5200 Lake Road., Merced, CA 95340, USA; 2 Ecology and Evolutionary Biology, Brown University, Box G-W, 80 Waterman Street, Providence, RI 02912, USA; 3 Center for Computational Biology, Brown University, Box G-W, 115 Waterman Street., Providence, RI 02912, USA

**Keywords:** convergent evolution, human adaptation, high altitude, domestication

## Abstract

Humans and their domestic animals have lived and thrived in high-altitude environments worldwide for thousands of years. These populations have developed a number of adaptations to survive in a hypoxic environment, and several genomic studies have been conducted to identify the genes that drive these adaptations. Here, we discuss the various adaptations and genetic variants that have been identified as adaptive in human and domestic animal populations and the ways in which convergent evolution has occurred as these populations have adapted to high-altitude environments. We found that human and domesticate populations have adapted to hypoxic environments in similar ways. Specific genes and biological pathways have been involved in high-altitude adaptation for multiple populations, although the specific variants differ between populations. Additionally, we found that the gene *EPAS1* is often a target of selection in hypoxic environments and has been involved in multiple adaptive introgression events. High-altitude environments exert strong selective pressures, and human and animal populations have evolved in convergent ways to cope with a chronic lack of oxygen.

This article is part of the theme issue ‘Convergent evolution in the genomics era: new insights and directions'.

## 1. Introduction

Environments shape the genetic landscape of the populations that inhabit them. As human populations have expanded across the world, they have encountered numerous novel environments, with associated changes in temperature and climate. One of the most challenging environments humans have encountered is high altitude. As elevation above sea level increases, a decrease in barometric pressure results in fewer oxygen molecules in the air, which causes hypoxia. Short-term exposure to high-altitude environments can cause acute altitude sickness, with symptoms including pulmonary and cerebral oedema (fluid accumulation in the lungs and brain). Long-term exposure to this environment can also cause pulmonary hypertension and is known to increase the risk of pregnancy complications including pre-eclampsia, which can be fatal [1]. Organisms that move into high-altitude environments develop a number of short-term adaptations, including elevated haemoglobin concentration, increased red blood cell count and higher resting ventilation. However, populations that have lived in high-altitude environments over many generations have been subjected to selective pressures and have adapted physiologically and genetically to live in these environments. In many cases, when the populations moved into high-altitude environments, they brought their domestic animals, including dogs, chickens and livestock. These animals were exposed to the same selective pressures as the human populations, and as a result, they show similar adaptations to hypoxic environments within the same timescales as their human cohabitants. Here, we summarize the history of adaptation to high altitude in human and domesticate populations worldwide, focusing specifically on convergent adaptations.

The term ‘convergent evolution' can have many definitions, but one definition concerns organisms that are distantly related developing the same or similar genetic adaptations that affect the organism's phenotype in the same way. Here, we focus on two specific cases of convergent evolution related to high-altitude adaptation. The first is the way in which human or animal populations from different geographical regions have developed similar genetic adaptations to living at high altitude. The second case is where different species show adaptation in the same biological pathways. Three regions of the world have been studied for populations' adaptation to life at high altitudes: the Andean Altiplano, Qinghai–Tibetan Plateau and the Ethiopian Highlands. In many human and animal populations, their physiological responses to hypobaric hypoxia have been measured, and their genomes have been compared with similar low-altitude populations in an attempt to identify the genetic variants that show signals of positive selection. The genes under selection are often part of the hypoxia-inducible factor (HIF) pathway, which governs the body's response to a lack of oxygen. We found that in many cases, the genotypic changes (either in the same gene or across multiple genes) differ between populations, but the phenotypic response is the same.

We performed a comprehensive literature search of studies of high-altitude adaptation in human and domestic animal populations. The majority of these studies focused on genome-wide selection scans, although a few studies looked instead at a set list of candidate genes. All genes that were identified by the authors of a study to be significant and likely important for high-altitude adaptation were included in this analysis. If a study included a functional analysis, we focused on the genes that were shown to have functional importance related to hypoxia. For Tibetans, which are the most-studied high-altitude human population, the genes with the strongest signals of selection are concordant across different genomic studies. Andean human populations, however, show less concordance between studies, as do Ethiopian human populations, which are the least-studied high-altitude human population. We then identified genes that were shared between populations, as well as genes involved in similar pathways. We also identified gene ontology (GO) terms for the biological processes associated with each of the genes (http://www.geneontology.org) using the PANTHER overrepresentation test (database and test both released on 10 October 2018) and used the PANTHER overrepresentation test to identify molecular function GO terms for each gene. As only four populations (Tibetan humans, Tibetan cattle, Tibetan goats and Andean humans) had overrepresented GO terms that were statistically significant with a Bonferroni correction, we used uncorrected *p*-values to identify GO terms to use for comparison. We then identified common GO terms across populations. A summary of the populations that have been studied and the genes that have been identified can be found in table 1, and a full list of GO terms that are shared between populations can be found in electronic supplementary material, S1.

**Table 1.** A summary of genes identified as selection targets for high-altitude adaptation in different populations worldwide. Bold genes are shared between populations, and genes in italics share GO terms that are common across multiple populations.

species	region	genes
human	Tibet	***EPAS1***, ***EGLN1***, ***PPARA***, *SLC52A3*, ***EDNRA***, ***PTEN***, *ANGPTL4, Cyp17A1, CYP2E1, HMOX2, CAMK2D, GRB2, ANKH,* RP11-384F7.2, HLA-DQB1/HLA-DPB1, *ZNF532*, KCTD12, *VDR, PTGIS, COL4A4, MKL1*, **HBB,** *MTHFR*
	Ethiopia	*VAV3, RORA*, SLC30A9, *COL6A1, HGF, BHLHE41, SMURF2, CASP1, CIC*, **LIPE**, *PAFAH1B3*, CBARA1, *ARNT2, THRB*, ***EDNRB***
	Andes	***EDNRA***, *VEGF, TNC, CDH1*, ***PRKAA1***, *NOS2A, BRINP3, SH2B1*, PYGM, *TBX5, DST*, SGK3, *COPS5*, ANP32D, *SENP1, PRDM1, PFKM*, ***EGLN1***
cow	Tibet	(Yak): ADAM17, ARG2, MMP3, CAMK2B, GCNT3, HSD17B12, WHSC1, Glul (Cattle): ***EGLN1***, *HIF3A*
	Ethiopia	*BDNF, TFRC*, ***PML***
horse	Tibet	*NADH6*
	Andes	***EPAS1***, *TENM2*, CYP3A cluster
pig	Tibet	*RGCC*, ***GRIN2B***, C9ORF3, GRID1, PLA2G12A, *ALB*, SPTLC2, *GLDC, ECE1, GNG2, PIK3C2G*
chicken	Tibet	SLC35F1, ***RYR2***
dog	Tibet	***EPAS1***, **HBB**, *AMOT, SIRT7, PLXNA4, MAFG, ENO3*, KIF1C, *KIF16B*, DNAH9, *NR3C2*, SLC38A10, ESYT3, ***RYR3***, *MSRB3*, ***CDK2***, *GNB1*
goat	Tibet	***EPAS1***, *SIRT1, ICAM1, YES1, JUP, CDK2*, ***EDNRA***, *SOCS2*, NOXA1, *ENPEP*, *KITLG*, ***FGF5***
sheep	Tibet	**FGF7**

## 2. High-altitude adaptations

### (a) Tibet

Tibetan populations have possibly been studied the most thoroughly for their adaptations to high altitude, based on the number of publications on the subject. At high altitudes (approx. 3000–4500 m), Tibetans have a high resting ventilation but low arterial oxygen content and low oxygen saturation—of the three populations who have lived at high altitudes for generations, they are the most hypoxic [2]. There is a correlation in women between higher levels of oxygen saturation and an increased number of surviving children, and alleles undergoing positive selection are associated with more positive pregnancy outcomes in Tibetan women [3], suggesting that selection is still acting on this population. Some Tibetans show an increase in haemoglobin concentration, but only at altitudes higher than 4000 m [2]. Genetic comparisons of Tibetan and Han Chinese individuals identified *EPAS1* (endothelial PAS domain protein 1), *EGLN1* (egl-9 family HIF 1) and *PPARA* (peroxisome proliferator-activated receptor alpha) [4–7], all of which are part of the HIF pathway. In some of these genes, the genetic mutation that may cause the physiological response has been identified [8]. For example, *EGLN1* is involved in HIF degradation, which triggers red blood cell production. A missense mutation in *EGLN1* results in a lack of elevated haemoglobin, at least under high-altitude conditions [9]. The *EPAS1* allele found in Tibetans was found to be associated with haemoglobin concentration as well [10]. The *EPAS1* variant in Tibetans shows the most differentiation from the Han Chinese [11] and shows more differentiation than that of any genetic variant between any two similarly closely related populations [12]. Surprisingly, the *EPAS1* haplotype found in Tibetans is only shared with a small number of Han Chinese and the Denisovan genome, which suggests that the Denisovan allele has a role in high-altitude adaptation in Tibetans. This is an example of adaptive introgression, in which gene flow between species or subspecies results in the introduction of a genetic variant that increases fitness [13–15]. A 3.4 kB deletion near *EPAS1* is found at high frequencies in Tibetans and is in strong linkage disequilibrium with the *EPAS1* haplotype, but it is extremely rare worldwide and may be a functional contributor to hypoxic tolerance in Tibetans [16]. A more recent study sequencing whole genomes identified a much larger haplotype block surrounding *EPAS1*, as well as additional candidate genes [17]. An expanded study of populations across the Himalayan region, including five ancient Himalayan genomes, suggests that most Himalayan populations derive a large proportion of their ancestry from a single ancestral population who adapted to high altitude [18]. However, admixture with other nearby populations has been detected [19–22], suggesting a complex demographic history of the region. Multiple gene variants that show signals of selection in modern Tibetans, including *EPAS1*, *EGLN1* and *SLC52A3* (solute carrier family 52 member 3), were also found in the ancient Nepalese genomes, which range in age from 1200 to 3100 years before present [18,23]. However, only some variants of these genes found in modern populations were found in the ancient genomes (for example, 6 of 26 in *EPAS1* and 11 of 21 in *EGLN1*), suggesting that selection for some of these gene variants has occurred recently.

Humans are not the only species that has adapted to this environmental niche in Tibet. The Tibetan Mastiff is a dog breed that was developed to live at high altitudes, and they show a lowered haemoglobin concentration compared with lowland Chinese native dogs [24]. A selection scan identified the target genes *EPAS1* and *HBB* (haemoglobin subunit beta), both of which have also been implicated in high-altitude adaptation in Tibetan human populations. Out of 16 genes that showed signals of positive selection, 12 were linked with hypoxia. Further exploration of *EPAS1* in Tibetan mastiffs showed four novel non-synonymous mutations in the gene, all of which are associated with decreased blood flow resistance [25]. A more recent study has suggested that the *EPAS1* variant in Tibetan Mastiffs is actually the result of adaptive introgression with Tibetan wolves [26]. An additional analysis focusing on the X chromosome identified the gene *AMOT* (angiomotin) as a target of selection, which is involved in blood pressure regulation [27].

In addition to dogs, numerous other domesticated animals living on the Tibetan Plateau show similar adaptations to the human populations. For instance, there are multiple pig populations living at high altitude in Tibet, all of which show signals of selection for variants of hypoxia-related genes [28]. Some genotypic changes, such as variants of *RGCC* (regulator of cell cycle) are shared between populations, while others, such as a *GRIN2B* variant (glutamate ionotropic receptor NMDA type subunit 2B) are unique to specific populations, demonstrating a combination of convergent evolution and novel variation to cope with the challenging environment of high altitudes. Tibetan chickens have an increased red blood cell count and blood oxygen affinity relative to other chicken populations [29], similar to Andean human populations, and a selection scan identified genes that had been positively selected and are involved in the calcium-signalling pathway, including *RYR2* (ryanodine receptor 2), which may be linked to high-altitude tolerance [30]. The Tibetan cashmere goat shows signals of selection in a number of genes that may be associated with hypoxia, including *EPAS1* [31]. Other breeds of goat living on the Tibetan Plateau show signals of positive selection in other genes, including *CDK2* (cyclin-dependent kinase 2) and *EDNRA* (endothelin receptor type A) [32]. Tibetan sheep breeds living at high altitude share a highly conserved functional variant in the promoter region of *FGF7* (keratinocyte growth factor 7), a gene that is involved in pulmonary diseases, suggesting that transcription modulation of the gene helps with survival in a hypoxic environment [33]. A study of Tibetan horse mitochondrial genomes identified multiple non-synonymous substitutions in the *NADH6* (ubiquinone oxidoreductase core subunit 6) gene, suggesting an involvement of energy metabolism in their adaptation to hypoxic conditions [34]. A comparison of the alpine yak genome to the cow genome showed increased divergence in gene families responsible for hypoxic stress, and hypoxia-associated genes like *ADAM17* (ADAM metallopeptidase domain 17) and *ARG2* (arginase 2) show signals of positive selection [35]. A more recent study of Tibetan cattle identified an association between an *EGLN1* variant and lowered haemoglobin concentration, and the adaptive variant was likely introgressed from hybridization with yaks [36].

### (b) Andes

The first population to be studied for their adaptation to high altitude was the Andeans, who live at 2500–4500 m. While Andeans thrive at high altitudes comparable to those that Tibetans experience, they have adapted to the hypoxic environment in different ways [37]. Compared with sea-level populations, they show a normal resting ventilation, but a slightly decreased oxygen saturation, increased haemoglobin concentration and a higher arterial oxygen content [2]. This is in contrast to Tibetan populations, who show no elevation in haemoglobin except at altitudes above 4000 m. A candidate gene study comparing Andean Native American populations to lowland Native American populations identified signals of positive selection for a number of genes, including *EDNRA*, *PRKAA1* (protein kinase, AMP-activated and alpha 1 catalytic subunit) and *NOS2A* (nitric oxide synthase 2A) [38]. In a follow-up genome-wide study, *EGLN1* (egl-9 family HIF 1) was also identified when only considering the genes in the HIF pathway [7]. Another genome-wide study of Andean high-altitude populations identified multiple genes under selection that are associated with cardiovascular function, including *BRINP3* (BMP/retinoic acid-inducible neural specific 3) [39]. This suggests that instead of selection targeting genes associated with the HIF pathway, as is the case with Tibetans, selection in Andeans instead has focused on modifications of the cardiovascular system to cope with high altitudes. Consistent with this hypothesis, a study of ancient Andean genomes dating to as early as 7000 years before present revealed selection in *DST* (dystonin), a gene involved in cardiovascular function [40]. Multiple studies comparing high-altitude individuals with and without chronic mountain sickness further identified additional genes that seem to protect against chronic disease at high altitude, including *ANP32D* (acidic nuclear phosphoprotein 32 family member D), *SENP1* (SUMO-specific peptidase 1) and *PRDM1* (PR/SET domain 1) [41,42].

Domestic animals from the Andes have also been studied for their adaptation to high altitudes. Llamas, as well as their close relatives the alpaca and vicuña, were adapted to high altitudes before they were domesticated and have a lower red blood cell count and haemoglobin levels relative to Andean humans at high altitude. However, it has been suggested that the smaller size and unique shape of camelid red blood cells better facilitate oxygen saturation [43]. Additionally, llama haemoglobin has a high oxygen affinity, such that oxygen saturation stays above 90% both at sea level and at high altitude [44]. Similarly, guinea pigs, which were also hypoxia-adapted prior to domestication, show a very limited increase in red blood cell count at higher altitudes and only a slight decrease in oxygen saturation at high altitudes compared with sea-level populations [45]. Even Andean chicken populations, which were introduced to the region following European contact (i.e. less than 500 years before present) have haemoglobin with a higher oxygen affinity, suggesting that adaptation to high altitude can occur within a population even in a relatively short period of time [46].

Investigation of the genetic changes underlying the physiological changes in Andean domesticates has been limited to studies of two species: alpacas and horses. Alpacas and other camelids share an ancient helix-loop-helix deletion from the HIF-1A protein with cetaceans and artiodactyls, which may contribute to their reduced hypoxic response [47]. A genomic study of feral Peruvian horses, which are descended from the horses the Spanish conquistadors introduced and now thrive at high altitudes, identified numerous genes showing signals of selection, including several associated with nervous system development as well as *EPAS1* [48].

### (c) Ethiopia

Ethiopian populations are the least studied for their adaptations to high altitude and are also more difficult to study. Ethiopian highlanders are not an isolated population like Tibetans and also show evidence of gene flow from outside of Africa [49], which can make it more difficult to identify adaptive variants. Unlike the Andeans and Tibetans, Ethiopians show no physiological response to high altitudes [2]. Their respiration, oxygen saturation and haemoglobin concentration are all similar to the values of humans at sea level. However, one study found that high-altitude Ethiopian populations exhibit higher haemoglobin levels at high altitude (compared with low altitude) and also identified differences in haemoglobin levels between closely related high-altitude Ethiopian populations living at the same altitude [49]. The effects of hypoxia may be lessened in these high-altitude populations, given that they live at a slightly lower altitude compared with Tibetans and Andeans (2500–3500 m). However, lowland populations living at high altitude would be expected to show elevated haemoglobin levels and increased respiration, demonstrating that Ethiopian highlanders do show some adaptation to high-altitude environments. A candidate gene study in Ethiopians identified multiple genes associated with the HIF pathway, including *VAV3* (vav guanine nucleotide exchange factor 3), all of which are distinct from those identified in Andeans and Tibetans [50]. A comparison between two populations, the Amhara who have lived at high altitudes for thousands of years and the Oromo who only moved into high-altitude environments in the past 500 years, identified selection on hypoxia-associated genes including *RORA* (RAR-related orphan receptor A) in the Amhara but no genes were identified in the Oromo, suggesting that they had not lived at high altitudes long enough to show signs of adaptation [51]. A more recent study of the Amhara and Oromo that made corrections to account for non-African admixture identified *BHLHE41* (basic helix-loop-helix family member E41), which is involved in the initiation of the hypoxic response via the HIF pathway, as the gene with the strongest selection signal in both populations [52]. This study also suggested that the Oromo may have acquired the adaptive *BHLHE41* variant through admixture with the Amhara, allowing them to adapt to high altitudes more rapidly. An additional three genes, *CIC* (capicua transcriptional repressor), *LIPE* (lipase E, hormone-sensitive type) and *PAFAH1B3* (platelet-activating factor acetylhydrolase 1b catalytic subunit 2), were identified using whole-genome sequencing with a subsequent demonstration of increased hypoxia tolerance in the orthologous genes in *Drosophila* [53]. A fourth gene, *EDNRB* (endothelin receptor type B), was shown to show increased hypoxia tolerance when knocked down in mice [54].

High-altitude cattle breeds living in Ethiopia show no elevated haemoglobin levels or red blood cell counts and a lower oxygen saturation relative to other cattle breeds living at high altitudes in the region [55], a similar adaptation to Ethiopian human populations [2]. Although oxygen saturation levels below 80% were reported as lethal for lowland cattle breeds in other studies, the high-altitude Ethiopian cattle breeds were thriving with a much lower oxygen saturation level of 68% [55]. A comparison of high-altitude and low-altitude Ethiopian cattle breeds showed high genetic differentiation between the populations for multiple genes associated with hypoxia, including *BDNF* (brain-derived neurotrophic factor), *TFRC* (transferrin receptor) and *PML* (promyelocytic leukemia), suggesting that these genes have been selected for increased hypoxia tolerance [56].

## 3. Discussion

### (a) Convergent evolution at the gene level

**Figure 1.**
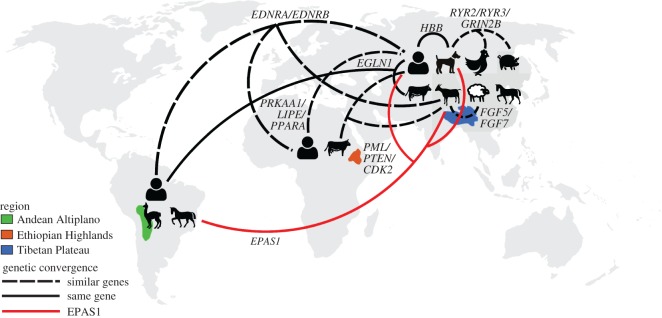
A map summarizing the genomic studies done on human and domesticate populations at high altitude, highlighting the convergent evolution between populations. The three high-altitude regions are highlighted, and the human and animal icons show the different species that have been studied in each region. Lines connecting populations indicate adaptation in the same or similar genes—solid lines indicate adaptation in the same gene, with EPAS1 highlighted in red, and dashed lines indicate similar genes that show adaptation across multiple populations. Pig and sheep icons from icons8.com, cow icon by Olivier Guin from thenounproject.com, chicken icon and goat icon by tan from onlinewebfonts.com and human icon by Dave Gandy from fontawesome.io, used under Creative Commons.

Although there is a considerable variation in how different human and animal populations have responded to high-altitude environments, there are several examples of convergent evolution across populations. A map of all populations studied and the convergent evolutionary connections between them is summarized in figure 1. For example, *HBB* shows the signals of positive selection both in human and in dog populations, and *EGLN1* was identified as adaptive in Tibetan and Andean human populations and in Tibetan human and cattle populations. *EDNRA* variants are also selected for in two human populations (Andeans and Tibetans) as well as in Tibetan goats. *EDNRB*, a closely related gene, was implicated in hypoxia tolerance in Ethiopian humans as well. Additionally, convergent evolution seems to have occurred in different genes involved in the same pathways in different populations. For example, *FGF5* in cashmere goats and *FGF7* in Tibetan sheep are both fibroblast growth factors, which can help protect against lung injury. *PML* in Ethiopian cattle, *PTEN* (phosphatase and tensin homolog) in Tibetan humans and *CDK2* in Tibetan goats are all involved with triggering apoptosis, which is a common response of cells exposed to hypoxic environments, either owing to hypoxic stress or owing to the accumulation of deleterious mutations as a result of hypoxia [57]. *PRKAA1* and *LIPE* in Ethiopian humans and *PPARA* in Tibetan humans are both associated with lipid metabolism, which can be negatively impacted by chronic hypoxia as it increases the risk of developing fatty liver disease [58]. Additionally, *RYR3* in Tibetan mastiffs, *RYR2* in Tibetan chickens and *GRIN2B* in Tibetan pigs are all associated with the regulation of calcium channels, which are important for a number of biological functions, including vasoconstriction [59].

*EPAS1* has been shown to be under positive selection in Tibetan humans, the Tibetan mastiff, feral Andean horses and cashmere goat populations living on the Tibetan Plateau. Interestingly, *EPAS1* is also one of the few genes that has been identified to be introduced through adaptive introgression, possibly from Denisovans into humans [12] and from Tibetan wolves to the Tibetan Mastiff [26]. In another instance of adaptive introgression, an *EGLN1* variant that is adaptive to high-altitude environments was likely introduced into Tibetan cattle populations through interbreeding with yaks [36]. Adaptive introgression is surprisingly a common component of high-altitude adaptation, likely because admixture with another species that is already adapted to an environmental niche is generally a more efficient way of acquiring adaptive variants than random, de novo mutation. Although not specifically an example of adaptive introgression, gene flow from the Ethiopian Amhara population likely introduced an adaptive *BHLHE41* variant to the Oromo, who migrated much more recently to high altitudes. This is especially true in cases such as that of the Tibetan Mastiff, where the adaptive *EPAS1* variant was not already segregating in the lowland population [26]. By interbreeding with the Tibetan wolf [60], which had lived at high altitudes for thousands of years prior to the arrival of the domestic dogs, Tibetan Mastiffs likely quickly acquired the genetic variants needed to thrive on the Tibetan Plateau.

*EPAS1* variants have also been identified as adaptive in numerous other animal species, including the Tibetan wolf [60], a Tibetan hot-spring snake [61], a rodent known as the plateau zokor [62], the High Himalayan frog [63] and the snow leopard [64]. It is surprising that despite a large number of genes implicated in high-altitude adaptation, *EPAS1* variants are involved so frequently. However, other examples of parallel evolution across distantly related taxa have been identified, and it has been suggested that there are genetic ‘hotspots’, where some genes are more likely to be modified in response to selective pressure than others [65,66]. Genes may be more likely to evolve if they have a larger effect size, a higher mutation rate, or a higher likelihood of mutations being beneficial [66]. It is possible that some characteristics of the *EPAS1* gene make it more likely to change in response to selective pressures or to be spread to other species through introgression.

### (b) Convergent evolution at the pathway level

**Figure 2.**
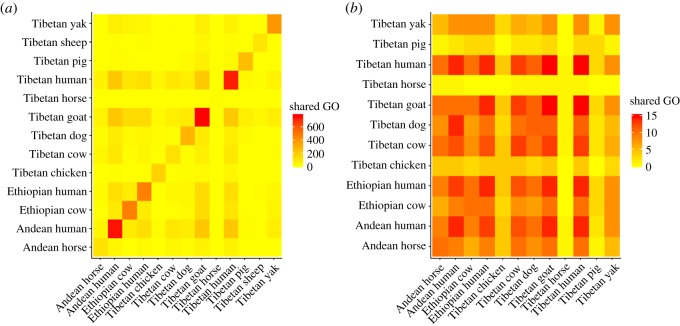
A summary of the GO terms shared between high-altitude populations. (*a*) The pairwise counts of all GO terms shared between populations. (*b*) The pairwise counts of common GO terms (found in at least half of the high-altitude populations sampled) shared between populations. Tibetan Horses are omitted from (*b*) because they did not have any of the common GO terms. (Online version in colour.)

At the level of biological function, many high-altitude populations share similar GO terms (figure 2; electronic supplementary material, S1). Here, we focus on terms that were shared across the majority of populations. Nine of the populations (Tibetan chickens, cattle, dogs, humans and yaks; Andean horses and humans and Ethiopian cattle and humans) shared three GO terms, all related to the hypoxia response: response to decreased oxygen levels, response to oxygen levels and response to hypoxia. Other GO terms that were overrepresented in at least half of the human and domesticate populations are related to development (anatomical structure morphogenesis, system development, multicellular organism development, muscle structure development, blood vessel morphogenesis and regulation of biological quality), responses to chemicals (response to chemical and cellular response to chemical stimulus) and stress (response to stress and response to oxidative stress), as well as regulation of transcription (regulation of transcription by RNA polymerase II) and nucleic acid production (positive regulation of nucleobase-containing compound metabolic process). These common GO terms suggest that while modifications to the response to hypoxia and other stressors are important for increased fitness in high-altitude populations, other pathways are also involved in helping improve fitness in these populations, including developmental changes (including changes to blood vessels and muscles), modulation of transcription and the responses of cells to stimuli. Interestingly, these GO terms are found across all three regions studied, emphasizing that convergent phenotypic changes occur in disparate populations as they adapt to high altitude, even if the specific genes that are selected for are different. A list of the genes identified in each population with the above GO terms can be found in electronic supplementary material, S2.

### (c) Phenotypic but not genotypic convergence

In many cases, when the same gene is selected for across multiple populations, we found that different single nucleotide polymorphisms (SNPs) have been implicated as adaptive in different populations. Therefore, while we see convergence in the genes under selection, the specific variants differ. This implies some redundancy in these genes, where multiple mutations can have the same effect. Several of the changes identified across populations in the HIF pathway result in decreased gene function or activity, and this decrease in function can likely be caused by a number of unique mutations. This phenomenon has also been identified in other cases of human adaptation. For example, the 32-base pair deletion in *CCR5* that promotes HIV1 immunity is found primarily in individuals of European descent [67], but other variants with a similar function have been identified in African populations [68]. Additionally, lactase persistence has arisen independently in multiple populations, with different adaptive variants arising in different regions of the world [69]. This suggests that this type of convergence, where different variants of the same gene have the same phenotypic effect, is a fairly common occurrence.

Although many of the same genes and pathways show similar signals of selection across species, the nature of the SNPs under selection seems to differ between humans and other species. In humans, nearly all of the adaptive mutations that have been identified seem to be outside of gene coding regions, but in domesticated species, there are multiple examples of non-synonymous mutations under selection. These include multiple genes (*EPAS1*, *HBB* and *AMOT*) in the Tibetan Mastiff [25–27], as well as *EPAS1* in cashmere goats [31], *NADH6* in Tibetan horses [34] and the modified *HIF1A* protein in alpaca and other camelids [47]. In other cases, however, including *FGF7* in Tibetan sheep [33] and *RYR2* in Tibetan chickens [30], regulatory regions have instead been a selection target. Generally, mutations in *cis*-regulatory regions are more likely to be selected for than mutations in coding regions, as they are less likely to have pleiotropic effects, which could be deleterious [70]. It is therefore unexpected that non-synonymous mutations selected in high-altitude populations are fairly common in domesticated species. However, the domestication process and ongoing animal husbandry exert strong selective pressures on domesticates, and the strength of selection can overcome the possible negative effects of selecting for mutations in the coding region [71].

### (d) Limitations of current studies

One caveat to this meta-analysis is that it can be difficult to identify specifically convergent genetic changes in these genes. The majority of these studies have focused on identifying regions that differ between related lowland and highland populations, rather than trying to identify functional changes and the effect they would have on an organism's phenotype. While some coding-region changes have been identified in Tibetan humans [9,16] and goats [31] and Andean humans [41], the majority of SNPs have been identified in non-coding regions, suggesting that gene regulation is affected. Additionally, few studies have attempted to identify genome-wide associations between genotype and phenotype [3,10] and only a handful of physiological studies have been conducted in domesticated populations, making it challenging to show convergence in genetic changes and their effects across species. It is also possible that selection is not acting on the physiological traits being measured (e.g. haemoglobin concentration and oxygen saturation), but these phenotypic changes are instead a result of selection for a different trait. However, we have identified convergent physiological changes between species, including increased oxygen affinity in Tibetan and Andean chickens and Andean humans and decreased haemoglobin concentration in Tibetan humans and dogs. To better identify the cases of convergent evolution and to determine if these candidate genes are actually being modified in parallel directions across populations, further exploration of the physiological and functional changes in high-altitude populations is needed to identify and characterize how genetic variants are driving this adaptation to high altitude.

Additionally, there is a bias in identifying genes that are part of the HIF pathway, which may be over-inflating the importance of hypoxia-associated genes for adaptation to high altitudes. Some studies have specifically examined hypoxia-associated genes only [38,56], while others identify a number of candidate genes but only focus on those related to hypoxia, despite the fact that other biological processes are important in high-altitude adaptation, as shown by the GO analysis. While adaptation to the reduced-oxygen environment seems to be important for the majority of high-altitude populations, an adaptation of the cardiovascular system to handle the increased heart rate and red blood cell count and changes to the placenta and other biological processes involved in pregnancy are also important to ensure that the population thrives at higher elevations. Additional adaptations would also be needed to cope with the colder temperatures and increased UV radiation at higher altitudes.

### (e) Future directions

Although many domesticates have been studied for their adaptations to high altitude, the majority of genetic studies have focused on Tibetan human and animal populations. More extensive genetic and phenotypic studies of Ethiopian human populations are needed to help further clarify the ways in which different populations adapt to higher altitudes. With a full understanding of the genetic variants that have been selected for in Tibetans, Andeans and Ethiopians, it will be possible to compare adaptations between populations who have lived at high altitudes for long periods of time (Tibetans and some Ethiopians) to those who have moved into high-altitude environments more recently (such as the Andeans). Cattle, chickens and other domesticates have also lived on the Andean Plateau and in the Ethiopian Highlands, and by studying the genes under selection in those populations, we gain a better understanding of how domesticates adapt to new environments, and whether these adaptations show phenotypic or genotypic convergence. Guinea pigs and camelids lived at high altitudes long before humans domesticated them, and it would be interesting to compare the adaptations of these species to those of species that were domesticated before they were exposed to hypoxic conditions, such as Andean chickens. Physiological analyses of domestic species should also be expanded, to allow for more direct comparisons between populations and to identify links between genotype and phenotype. Finally, while some ancient DNA studies of high-altitude populations have been conducted [18,40], further study of ancient human and animal populations at high altitude can reveal how these populations adapted to high altitudes over time. By continuing to study the populations that live and thrive at high altitudes, we gain a better understanding of the convergent ways in which species adapt to new environments.

The study of human and domesticate populations in Tibet, the Andes and Ethiopia has enabled us to begin to understand how species adapt to life at high altitude and has also provided insight about the nature of convergent evolution. High altitude is an excellent natural experiment for studying convergent evolution because it has an ecological context, and hypoxia affects all species living at high altitudes. These regions have been populated with many different species, including wild animals, domesticates and humans, and in some cases (such as in humans and in cattle), we have parallel examples of the same species immigrating to high-altitude environments in different regions of the world. This enables us to capture the breadth of adaptation to a single environment. We have found that in some cases, convergent evolution occurs in specific genes (such as *EPAS1*), although the specific variants involved in adaptation may differ across populations. Additionally, although some pathways (such as the hypoxia response) seem to be more commonly involved in adaptation to a common environment, different populations follow different routes to become adapted to their environment. We found that in many cases, the phenotypic outcome and fitness outcome are the same despite genotypic differences, suggesting that convergent evolution can occur in a multitude of ways. With deeper genetic and phenotypic studies of modern and ancient high-altitude populations, as well as a functional assessment of the putatively selected variants, we can more fully characterize the nature of genetic convergence as populations worldwide have adapted to life at high altitude.

## Supplementary Material

Shared gene ontology terms

## Supplementary Material

Genes in most common gene ontology terms

## Data accessibility

This article has no additional data.
